# Biological Aging Predicts Vulnerability to COVID-19 Severity in UK Biobank Participants

**DOI:** 10.1093/geroni/igab046.1285

**Published:** 2021-12-17

**Authors:** Chia-Ling Kuo, Luke Pilling, Janice Atkins, Jane Masoli, Joao Delgado, George Kuchel, David Melzer, Morgan Levine

**Affiliations:** 1 University of Connecticut Health, Farmington, Connecticut, United States; 2 University of Exeter, Exeter, England, United Kingdom; 3 University of Exeter Medical School, University of Exeter, England, United Kingdom; 4 University of Exeter Medical School, Exeter, England, United Kingdom; 5 Yale University, New Haven, Connecticut, United States

## Abstract

Age and disease prevalence are the two biggest risk factors for COVID-19 symptom severity and death. We therefore hypothesized that increased biological age, beyond chronological age, may be driving disease-related trends in COVID-19 severity. Using the UK Biobank England data, we tested whether a biological age estimate (PhenoAge) measured more than a decade prior to the COVID-19 pandemic was predictive of two COVID-19 severity outcomes (inpatient test positivity and COVID-19 related mortality with inpatient test-confirmed COVID-19). Logistic regression models were used with adjustment for age at the pandemic, sex, ethnicity, baseline assessment centers, and pre-existing diseases/conditions. 613 participants tested positive at inpatient settings between March 16 and April 27, 2020, 154 of whom succumbed to COVID-19. PhenoAge was associated with increased risks of inpatient test positivity and COVID-19 related mortality (ORMortality=1.63 per 5 years, 95% CI: 1.43-1.86, p=4.7x10E-13) adjusting for demographics including age at the pandemic. Further adjustment for pre-existing disease s/conditions at baseline (OR_M=1.50, 95% CI: 1.30-1.73 per 5 years, p=3.1x10E-8) and at the early pandemic (OR_M=1.21, 95% CI: 1.04-1.40 per 5 years, p=0.011) decreased the association. PhenoAge measured in 2006-2010 was associated with COVID-19 severity outcomes more than 10 years later. These associations were partly accounted for by prevalent chronic diseases proximate to COVID-19 infection. Overall, our results suggest that aging biomarkers, like PhenoAge may capture long-term vulnerability to diseases like COVID-19, even before the accumulation of age-related comorbid conditions.

